# A systematic review and meta-analysis on effect of spinal mobilization and manipulation on cardiovascular responses

**DOI:** 10.1142/S1013702520500122

**Published:** 2020-08-06

**Authors:** Charu Gera, Manoj Malik, Jaspreet kaur, Minaxi Saini

**Affiliations:** 1 Department of Physiotherapy, Guru Jambheshwar University of Science and Technology, Hisar, Haryana, India; 2 Mother Teresa Saket College of Physiotherapy, Saket, Panchkula, Haryana, India; jaspreet_malik16@yahoo.co.in

**Keywords:** Spinal manipulations, spinal mobilizations, cardiovascular responses, blood pressure, heart rate

## Abstract

**Background:**

Spinal pain or misalignment is a very common disorder affecting a significant number of populations resulting in substantial disability and economic burden. Various manual therapeutic techniques such as spinal manipulations and mobilizations can be used to treat and manage pain and movement dysfunctions such as spinal mal-alignments and associated complications. These manual therapeutic techniques can affect the cardiovascular parameters.

**Objective:**

The objective of this systematic review and meta-analysis is to assess the effect of spinal manipulation and mobilization on cardiovascular parameters.

**Methods:**

We conducted a systematic review and meta-analysis to assess the effects of spinal mobilization and manipulation on cardiovascular responses. Mean changes in Systolic Blood Pressure (SBP), Diastolic Blood Pressure (DBP) and Heart Rate (HR) were primary outcome measures. RevMan 5.3 software was used for the meta-analyses. Quality of the included studies was assessed by PEDro Rating scale. Risk of bias was assessed by Cochrane collaboration tool of risk of bias.

**Results:**

Results of meta-analysis showed that there was statistically significant decrease in SBP (MD=−4.56, 95% CI=−9.20, 0.08; p≤0.05) with moderate heterogeneity (I2=75%, p<0.0002) in experimental group as compared to control group. There was statistically non-significant decrease in DBP (MD=−1.96, 95% CI=−4.60, 0.69; p=0.15) with high heterogeneity (I2=91%, p<0.00001), Change HR was statistically non-significant (MD=−0.24, 95% CI=−3.59, 3.11; p=0.89) with moderate heterogeneity (I2=60%, p=0.01). Exclusion of short duration studies in sensitivity analysis revealed a statistically significant change in DBP (MD=−0.94, 95% CI=−1.85, −0.03; p=0.04). However, the result was statistically non-significant for HR after sensitivity analysis.

**Conclusion:**

Spinal manipulations and mobilizations may result in significant decrease of systolic as well as diastolic Blood Pressure.

## Introduction

Manual therapy techniques, like spinal mobilizations, high-velocity, low-amplitude manipulations and mobilization with movement are frequently used by physiotherapists along with various therapeutic exercises to treat or manage spinal pain and movement dysfunction.^1,2,3^ Spinal mobilizations are referred as “graded passive, oscillatory movements applied to the spine that moves it to the end of its available range”.^3^ These mobilizations are performed within the normal range of motion in such a way that it may be controlled by the patient, whereas manipulations are rapid movement at the end of the range of movement that cannot be controlled by the patient.^4^ Although both the techniques are different in application, still the main emphasis of both techniques is continuous assessment and evaluation.^1^ According to the findings of assessment and evaluation, spinal manipulative therapies are applied at varying speed and amplitude.^3^

Spinal manipulative therapies are mainly indicated to alleviate spinal pain and correct spinal malalignment.^1^ Spinal pain is a very common disorder affecting a significant number of populations resulting in substantial disability and economic burden. It is estimated that approximately 54–80% population suffer from spinal pain at any stage of their life.^5^ Research evidence suggests that workplace physical and psychosocial factors also contribute to the development of spinal pain. Gender, occupation, emotional problems, smoking, poor job satisfaction, awkward posture and poor work environment may also be associated with spinal pain.^6^

Spinal malalignments (such as scoliosis) are mainly caused by body’s abnormal posture, asymmetries in bone growth and abnormalities of neuromuscular system. Asymmetrical load applied to vertebral axis is the main cause of development and progression of spinal deformity. Altered biomechanics, weakness of abdominal muscles, joint laxity and increased extensibility of soft tissues can be risk factors for the progression of spinal malalignments.^7^ As a response, body progressively attains compensatory mechanism from other flexible parts of spine to preserve the spine posture. This puts additional stress over the musculoskeletal system and further leads to pain.^8^ Literature suggests that psychosomatic symptoms such as stress, anxiety and depression are strongly associated with spinal pain and malalignment.^9^ Spinal pain, mainly in cervical region, is strongly associated with migraine and severe headache with prevalence rate of 15.1%. Other associated symptoms are spine stiffness, headache, numbness, dizziness, sleeping difficulties, fatigue and memory as well as cognitive deficits.^6^

Evidence shows that spinal malalignment of cervical spine (especially C1 vertebra) can potentially injure, impair and compress brainstem neural pathways which is the regulatory centre of cardiovascular functions. Changes in the anatomical position of atlas [C1] and connected chain result in circulatory changes of vertebral artery. These circulatory abnormalities around the atlas vertebra and posterior fossa of brain have significant correlation with worsening of hypertension.^11^ Involvement of thoracic spine alone or in combination with lumbar spine results in cardiovascular and respiratory complications.^10^

Therefore, it may be suggested that the spinal manipulations, especially of cervical region can affect heart rate and blood pressure. The primary mechanism of these benefits can be parasympathetic stimulation.^3^ SMT of cervical spine may directly stimulate the parasympathetic flow via brain stem or indirectly through the stimulation of carotid sinus which further stimulates the brain stem via nucleus tractus solitaries (NTS).^11,12^

The existing research literature regarding effect of spinal manipulation and mobilization on cardiovascular parameters is still ambiguous. Studies corroborate^11,13,14,15,16,17,18,21,22,23,26,27,28^ as well as contradict^12,19,20,24,25^ the effects of manual therapy on cardiovascular parameters. These studies also have various methodological flaws. Further, small sample size of these studies limits the generalizability of their results.

Till date, no systematic review and meta-analysis has been done to assess the effect of spinal manipulation and mobilization on these cardiovascular parameters. Therefore, the rationale of this systematic review and meta-analysis is to include good quality randomized control trial (RCT’s), on this subject matter, so that a conclusion can be drawn on the basis of the collective inference of these RCTs.

## Methodology

### Search strategy and selection criteria

This systematic review and meta-analysis was developed according to the guidelines of the Preferred Reporting Item for Systematic Reviews and Meta-Analyses (PRISMA), 2015. The protocol of this systematic review and meta-analyses was registered under “The International Prospective Register of Systematic Reviews” (PROSPERO) with identification number **CRD42019124114.**

A comprehensive electronic database search for eligible trials (from inception to January 2019) was done in the Cochrane library (Cochrane Central Register of Controlled Trials) and PubMed. Reference lists were also examined to identify the articles not captured in the electronic database search. The research was restricted to RCT’s, done on humans and reported in English language using keywords spinal manipulation, spinal mobilization, vertebral adjustment, Mulligan approach, Maitland approach, blood pressure, heart rate, pulse rate, pulse oximetry, electrocardiogram and cardiovascular responses as according to Participant Intervention Comparison Outcome (PICO) strategy. During the search, Medical subject Headings (MeSH) terms, related keywords and “Boolean operators (‘OR’ and ‘AND’) using ‘Advanced’ search options’ were included. (*Search strategy as supplementary material 1*) RCTs that evaluated atleast one cardiovascular outcome such as BP or HR during or immediately following spinal manipulations and mobilizations were included. Reviews, systematic reviews, meta-analysis, case reports, editorials and letters were excluded. EndNote software (version X7.7) was used to remove the duplicate records of electronic databases. Two investigators (CG and MM) screened the titles and abstracts of the identified records. This was followed by full text screening performed independently by two investigators (CG, JK).

Inconsistencies were resolved by discussion among all the authors (CG, MM, JK and MS). If the study data was not available, corresponding researcher or the first researcher listed in the included articles was contacted to provide the missing data.

### Data extraction and quality assessment

Two investigators (CG and JK) independently extracted the data as per study objectives. Extracted information was then compared and discrepancies such as unclear or missing data presentation were resolved by discussion among the authors.

To assess the efficacy of treatment, mean change in blood pressure (BP) and heart rate (HR) was considered as the primary outcome. Missing data of standard deviation were imputed using correlation coefficient for change from baseline. Chi ($X^{2}$) test and $I^{2}$-statistic (degree of heterogeneity) were used to assess the Heterogeneity of the studies. Heterogeneity of “0–25% was considered as low heterogeneity, 26–75% as moderate heterogeneity and 76–100% as substantial heterogeneity” in $I^{2}$ test. Sensitivity analysis was also done in case of moderate or substantial heterogeneity. Review Manager (RevMan, version 5.3) software was used to perform the meta-analysis.

The PEDro rating scale was used to assess methodological quality of each study. The PEDro rating scale is a eleven-point scale which is used to evaluate the internal quality and validity of the randomized control trials. A score of 6 or more is considered as high quality; while a score of 4–5 is considered as fair (4–5) and 3 or below as poor quality trial.

Risk of bias was evaluated using Cochrane Collaboration’s modified tool. This assessment tool consists of seven primary sources for bias: “random sequence generation, allocation concealment, selective reporting, blinding of participants and personnel, blinding of outcome assessment, incomplete outcome data and other sources of bias”. These were evaluated independently by the authors to classify the risk of bias as a “high risk”, “low risk” or “unclear risk”.

Randomized controlled trials investigating the effect of spinal mobilizations and manipulations performed at any region of spine were grouped together for meta-analysis. Outcome measures were heart rate and systolic as well as diastolic blood pressure. Subgroup analysis of these outcome measures was performed on healthy individuals and patients of spinal pain or hypertension. The mean differences (MD) with standard deviations (SDs) of change in systolic blood pressure (SBP), diastolic blood pressure (DBP) and HR were calculated. Continuous variables were evaluated using confidence interval (CI) at 95% and weighted mean differences (WMD). Results of all eligible studies were considered as statistically significant at p≤0.05. The forest plots and funnel plots were generated using the Review Manager (RevMan, version 5.3) software. Further, sensitivity analysis was done in case of moderate or high heterogeneity. Sensitivity analysis was also performed by exclusion of short duration studies as well as studies with high weightage to evaluate the effect of these studies on outcome measures.

## Results

**Fig. 1.**
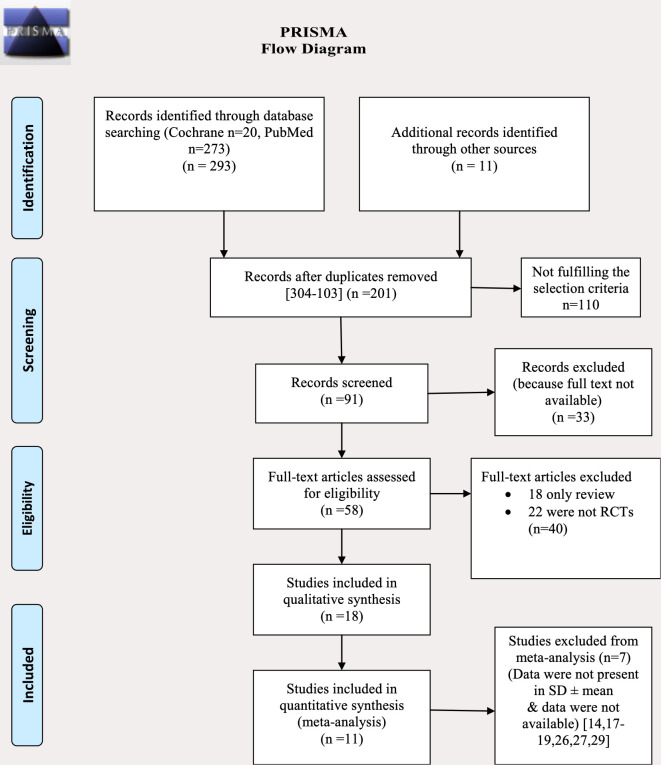
Flow diagram showing selection of studies.

**Study Selection.** A total of 304 articles were retrieved from the database searches, of which 18 met the selection criteria. Seven articles were excluded from the meta-analysis as required data couldn’t be retrieved. The details of the study selection have been represented in Fig. 1. The remaining 8 out of 11 articles comprising 141 participants in treatment group and 134 participants in control/placebo group were included for the meta-analyses of blood pressure. 9 out of 11 articles comprising of 147 participants in treatment group and 143 participants in control/placebo group were included for the meta-analysis of heart rate.

**Study characteristics.** Table 1 summarizes the characteristics of the included studies. Most of the studies were done in USA (nine)^11,12,19,21,22,23,25,27,28^ followed by Australia (two),^17,18^ Spain (two),^13,24^ Canada (two).^14,16^ One study each was conducted in Brazil,^15^ France^16^ and Malaysia.^20^ Only randomized control trials, either on healthy population or symptomatic population (such as subjects with spinal pain or hypertension), that compared the intervention to either a placebo or a control group were included.

**Table 1.** Major characteristics of included studies.

Art.	Author	No. of participants	Characteristics of participants	Study location	Study duration	Treatment	Outcome measures	Findings
1	Bakris *et al.*^11^	n=50, Treatment group (n=25) and control group (n=25).	Spinal (atlas vertebrae) malalignment with stage 1 hypertension	Barrington, USA	8 weeks	C1 correction	BP and pulse rate	Reduction in BP in treatment group. Pulse rate was not reduced.
2	Ward *et al.*^12^	n=48, 12 each, left head turn control, no contact, left atlas, right atlas.	Healthy individuals	Pasadena, United States	2 consecutive days	C1 manipulation	BP,ECG and pulse oximetry	No significant difference between experimental and control group.
3	Touche *et al.*^13^	n=48, experimental or placebo groups (n=24 each).	Cervical spine pain and cranio-facial pain	Madrid, Spain	8 months	Upper cervical mobilization	Pain, breathing rate and HR.	Decrease pain and increase breathing rate and HR in experimental gp.
4	Yates *et al.*^14^	n=21, active, placebo and no treatment control (n=7 each).	Thoracic spine pain, hypertensive and anxiety	Toronto, Ontario, Canada	6 weeks	T1–T5 Adjustment	BP and anxiety.	Decrease BP and anxiety in experimental group.
5	Reis *et al.*^15^	Women with Fibromyalgia (n=10) and healthy women (n=10).	Thoracic spine pain	Brazil	10 weeks	PA and central thoracic Maitland mob.	Pain, HR and RR	Improve HR in experimental group.Pain was not reduced.
6	Younes *et al.*^16^	n=22, Sham group (n=7) and SMT (n−10). Rest were left.	Mechanical Low back pain (lumbar spine pain)	France	6 months	HVLA thrust, lumber mobilization.	Pain, systolic BP and ECG.	Reduced HR and pain in SMT group. No effect on BP and ECG.
7	Vicenzino *et al.*^17^	n=24, treatment, placebo and no treatment group.	Healthy individuals	Brisbane, Australia	2 months	Lateral cervical glide	BP, RR and HR	Increase BP, HR and RR in treatment group as compared toplacebo group.
8	Farthing *et al.*^18^	n=30, rib raising, placebo and control treatment (n=10 each).	Asymptomatic and healthy participants	Melbourne, Australia	8 weeks	slow rib raising	HR, RR, BP and pain pressure threshold (PPT)	Increase RR, DBP and PPT in rib raising treatment as compared to placebo and control group.
9	Goertz *et al.*^19^	n=51, spinal manipulation (n=24) and sham gp (n=27).	Hypertensive patients	Davenport, Iowa, USA.	6 weeks	spinal manipulation of upper cervical	BP	No significant difference between SMT and Sham group.
10	Win *et al.*^20^	n=20, normotensive and neck pain patient group (n=10 each).	healthy volunteer and neck pain patients (cervical spine pain)	Malaysia	3 weeks	Spinal manipulation of upper and lower cervical spine.	Pain, BP and HR	Decrease in pain SBP in upperand lower cervical in treatment gp as compared to control.
11	Yung *et al.*^21^	n=39, treatment and placebo gp.	Healthy participants	Los Angeles, USA	3 months	AP glide at C6	BP and HR.	Decrease in systolic BP and HR in treatment gp.
12	Ward *et al.*^22^	n=50, SMT gp and control gp. (n=25 each)	Hypertensive individuals	Pasadena, TX, USA	3 months	Upper thoracic T1-4 SMT	ECG, BP and pulse oximetry.	Lower left pulse oximetry in SMT. Slight decrease in BP in SMT gp.
13	Yung *et al.*^23^	n=44, treatment and control group. (n=22 each).	Healthy, pain free participants	United States	2 years	PA mobilization at cervical region	BP, HR and pain	Decrease in SBP in treatment group and increase SBP in control group. No change in HR.
14	Valenzuela *et al.*^24^	n=37, Sham (n=19) & SMT (18) group.	Healthy individuals	Madrid, Spain	12 weeks	spinal manipulation therapy	HR, handgrip	Significant decrease in HR in SMT group than sham group.
15	Ward *et al.*^25^	n=36, treatment, placebo and control group. (n=12 each).	Thoracic spine pain participants	Pasadena, TX, USA.	3 months	T1-4 manipulation	ECG, BP and pulse oximetry.	No significant difference between treatment, placebo and control group.
16	Roy *et al.*^26^	n=51, pain gp (sham, treatment, n=20) and pain free (sham, treatment and control) gp. (n=31).	Spinal pain participants and Healthy individuals	Canada	4 months	spinal manipulation therapy at L5	Heart rate variability (HRV)	HRV decreased in sham and treatment group than control group
17	Roffers *et al.*^27^	Experimental group (n=99), control (n=95) and placebo (n=96).	Healthy participants and hypertensive participants	USA	8 weeks	T1-5 spinal manipulation	BP & pulse rate	BP and pulse rate was decreased in experimental group as compared to control and placebo group.
18	McMasters *et al.*^28^	n=24, prehypertensive and hypertensive stage 1 group (n=12 each).	Spinal pain patients and prehypertensive and hypertensive patients	Spartanburg, SC, USA.	1 year	spinal manipulation	BP	BP was decreased in hypertensive stage 1 patient group.

Eight out of the total 18 studies applied either spinal mobilization or manipulation to the cervical spine, six to the thoracic spine and two to the lumbar spine. One study applied manipulation to ribs while manipulation on whole spine was performed in two studies. All studies measured changes in cardiovascular responses (blood pressure and heart rate) during or immediately after the intervention.

**Table 2.** Assessment of quality of studies by PEDro scoring.

	1	2	3	4	5	6	7	8	9	10	11	Total
Articles	Specified eligibility criteria	Random allocation	Concealed allocation	Similar baseline	Subjects blinding	Therapists blinding	Assessors blinding	Measures of key outcomes from more than 85% of subjects	Intention to treat analysis of one key outcome	Statistical comparisons between-group of at least one key outcome	Variability for at least one key outcome	
1. Bakris *et al.*^11^	Yes	Yes	Unclear	Yes	Yes	Unclear	Unclear	Yes	Yes	Yes	Yes	8/11
2. Ward *et al.*^12^	Yes	Yes	Yes	Yes	No	No	Yes	Yes	Yes	Yes	Yes	9/11
3. Touche *et al.*^13^	Yes	Yes	Yes	Yes	Yes	No	Yes	Yes	Yes	Yes	Yes	10/11
4. Yates *et al.*^14^	Yes	Yes	Yes	Yes	Yes	Yes	Yes	Yes	Yes	Yes	Yes	11/11
5. Reis *et al.*^15^	Yes	Yes	Yes	Yes	No	No	No	Yes	Yes	Yes	Yes	8/11
6. Younes *et al.*^16^	Yes	Yes	Yes	Yes	Yes	No	Yes	Yes	Yes	Yes	Yes	10/11
7. Vicenzino *et al.*^17^	Yes	Yes	Yes	Yes	No	No	No	Yes	Yes	Yes	Yes	8/11
8. Farthing *et al.*^18^	Yes	Yes	Yes	Yes	Yes	No	Yes	Yes	Yes	Yes	Yes	10/11
10. Goertz *et al.*^19^	Yes	No	Yes	Yes	Yes	No	Yes	Yes	Yes	Yes	Yes	9/11
11. Win *et al.*^20^	Yes	Yes	Yes	Yes	Yes	Yes	Yes	Yes	Yes	Yes	Yes	11/11
12. Yung *et al.*^21^	Yes	Yes	Yes	Yes	Yes	Yes	Yes	Yes	Yes	Yes	Yes	11/11
13. Ward *et al.*^22^	Yes	Yes	Yes	Yes	Yes	No	Yes	Yes	Yes	Yes	Yes	10/11
14. Yung *et al.*^23^	Yes	Yes	Yes	Yes	Yes	Yes	Yes	Yes	Yes	Yes	Yes	11/11
15. Valenzuela *et al.*^24^	Yes	Yes	Yes	Yes	Yes	Yes	Yes	Yes	Yes	Yes	Yes	11/11
17. Ward *et al.*^25^	Yes	Yes	Yes	Yes	Yes	No	Yes	Yes	Yes	Yes	Yes	10/11
18. Roy *et al.*^26^	Yes	Yes	Yes	Yes	Yes	No	No	Yes	Yes	Yes	Yes	9/11
19. Roffers *et al.*^27^	Yes	Yes	Yes	Yes	Yes	No	Yes	Yes	Yes	Yes	Yes	10/11
20. McMasters *et al.*^28^	Yes	Yes	Yes	Yes	Yes	No	No	No	Yes	Yes	Yes	8/11

**Quality assessment.** Table 2 summarizes the quality of the included studies. All the studies ranked high on PEDro rating scale. 5 out of 18 studies scored the highest score of 11.^14,20,21,24,25^ 6 studies scored 10,^13,16,18,22,25,27^ 3 studies scored 9^12,19,26^ and 4 studies scored 8.^11,15,17,28^ Therefore, it can be inferred that all the included studies were of good quality.

**Fig. 2.**
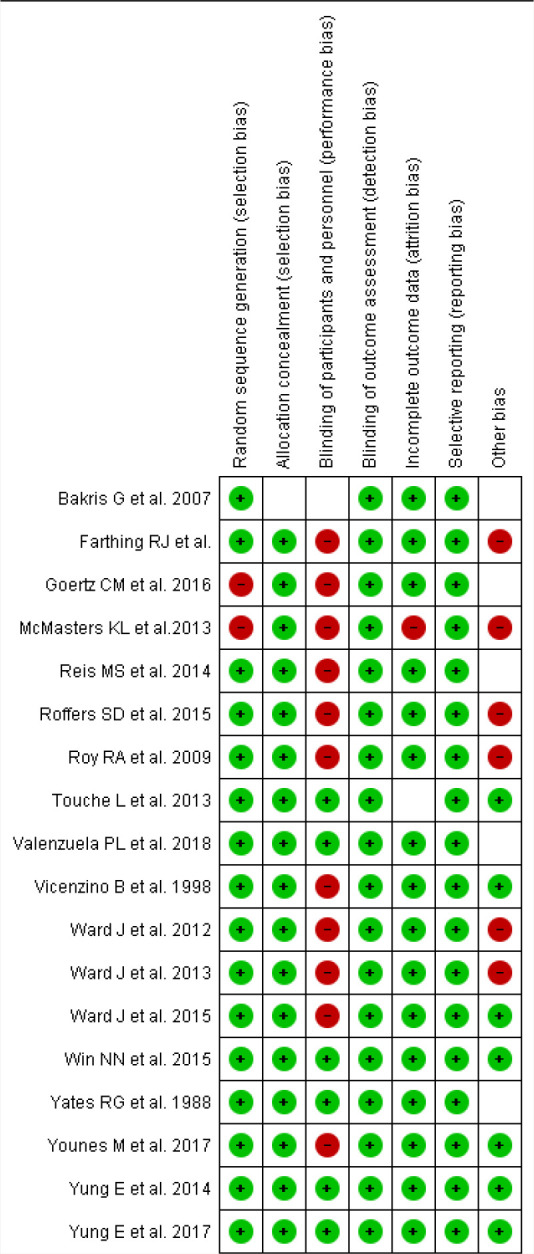
Risk of bias summary. Studies in green or + are at low risk of bias. Studies in red or − are at high risk of bias. Studies in blank are at unclear risk of bias.

**Risk of bias.** Risk of bias of the included studies is summarized in Fig. 2. “Random sequence generation” was described adequately in 16 studies.^11,12,13,14,15,16,17,18,21,22,23,24,25,26,27,28^“Allocation concealment” was done in maximum number of studies.^12,13,14,15,16,17,18,19,20,21,22,23,24,25,26,27,28^ “Blinding of participants and personnel” were described in six studies.^13,14,16,20,21,25^ The overall risk of bias was low in all the included studies.

**Fig. 3.**
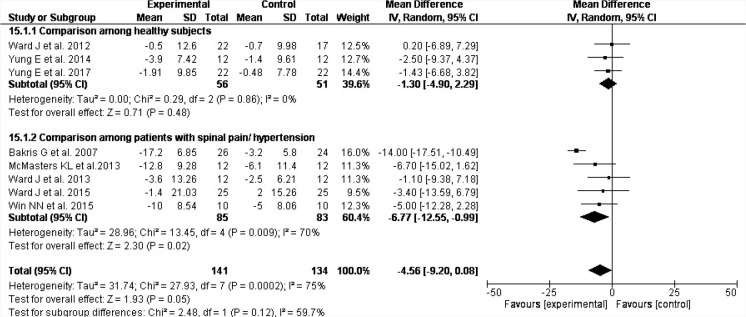
Comparison of systolic blood pressure using forest plot and subgroup analysis.

**Meta-analysis.** In primary outcome analysis of the studies, eight articles were included for the meta-analysis of BP. Spinal mobilization and manipulation resulted in a statistically significant reduction of SBP (MD=−4.56, 95% CI=−9.20, 0.08; p=0.05). However, the heterogeneity of the data was moderate ($I^{2}=75$%, p<0.0002) (Fig. 3). Additionally, subgroup analysis revealed that the reduction in SBP was more in patients with spinal pain and/or hypertension (MD=−6.77, 95% CI=−12.55, −0.99; p=0.02). There was statistically non-significant decrease in diastolic blood pressure (DBP) of experimental group as compared to control and placebo group with (MD=−1.96, 95% CI=−4.60, 0.69; p=0.15). However, the data was highly heterogeneous ($I^{2}=91$%, p<0.00001) (Fig. 5).

**Fig. 4.**
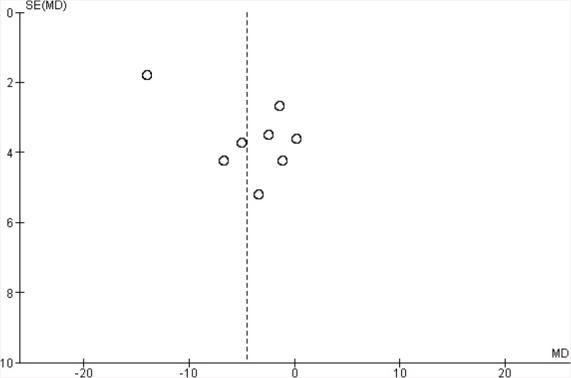
Funnel plot showing no publication bias in systolic blood pressure.

**Fig. 5.**
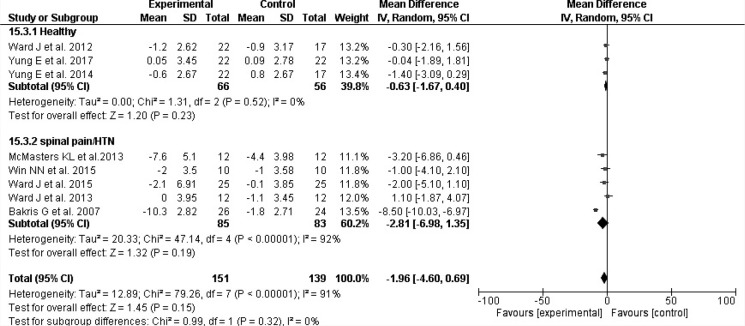
Comparison of diastolic blood pressure using forest plot and subgroup analysis.

Nine articles were included for the meta-analysis of HR. There were statistically non-significant changes in heart rate with (MD=−0.24, 95% CI=−3.59, 3.11; p=0.89) and moderate heterogeneity of $I^{2}=60\%$, p=0.01 (Fig. 7).

***Sensitivity analyses.*** Exclusion of studies with maximum weight has not affected the results for DBP and HR. But exclusion of one study has reduced the heterogeneity to 0% for HR. However, the result was statistically non-significant. (*Fig. S1 available as supplementary material* 2). Further, the sensitivity analysis was also done by omitting short duration studies. This revealed that diastolic blood pressure reduced significantly in experimental group as compared to control/placebo group (*Fig. S2 as supplementary material* 3) for long duration studies with (MD=−0.94, 95% CI=−1.85, −0.03; p=0.04). Exclusion of one study with maximum weight and another with smallest duration has reduced the heterogeneity to 0% and result was still significant for SBP. (*Fig. S3 available as supplementary material* 4).

## Discussion

Spinal pain and malalignment mainly occur due to structure deterioration, altered biomechanics and abnormal posture.^7^ Workplace physical and psychosocial factors, emotional problems, smoking, poor job satisfaction, awkward posture and poor work environment can be the possible risk factors for spinal pain and malalignment.^6^ This leads to various musculoskeletal, psychosomatic, cardiovascular and respiratory dysfunctions which affect the functional capacity of the patient as well as quality of life.^7,8,10^

In this meta-analysis, spinal manipulation and mobilization resulted in statistically significant reduction in SBP. Therefore, it can be used as an adjuvant therapy for the management of hypertension. These findings are supported by many previously published researches.^11,13,14,15,16,17,18,21,22,23,26,28^ However, some of the previous researches are contradictory to our results.^12,19,20,24,25^ Further, this meta-analysis also showed a statistically non-significant change in DBP and HR.

Sensitivity analysis was performed for diastolic blood pressure and heart rate. There was significant reduction in diastolic blood pressure after exclusion of short-term duration studies. Therefore, treatment duration and dosage may be attributed as an important factor. However, the results were statistically non-significant for HR.

Decrease in BP after application of spinal manipulative therapy and mobilization can be attributed to the influence on autonomic nervous system.^13,16^ The physiological mechanism behind the reduction of blood pressure can be neural input of spinal mobilization via pontomedullary reticular formation (PMRF) and contralateral intromediolateral cell column (IML) which relaxes the whole vasculature.^27^

Changes in the cardiopulmonary parameters such as blood pressure, heart rate and respiratory rate may be due to stimulation of sympathetic fibers through spinal mobilizations and multi-system, centrally co-ordinated response.^3^

This meta-analysis and systematic review corroborates that spinal manipulations and mobilizations are effective in decreasing blood pressure. Manipulations and mobilizations may be helpful in managing hypertension and its associated complications.

Meta-analysis revealed that there were moderate amount of heterogeneity in case of systolic blood pressure and heart rate. There was a substantial heterogeneity in case of diastolic blood pressure. Sensitivity analysis was performed to see the various sources of heterogeneity. Heterogeneity was reduced after sensitivity analysis. This further increases the reliability of our results.

**Fig. 6.**
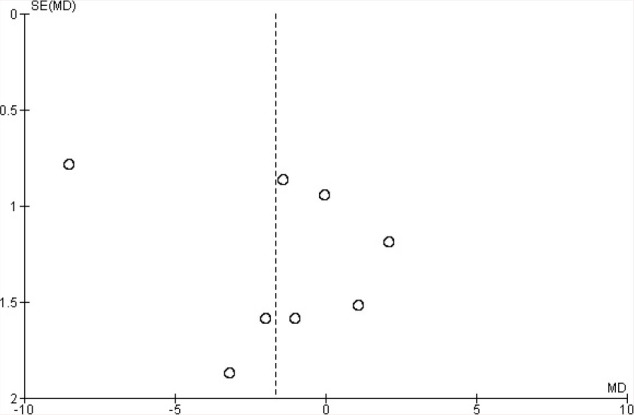
Funnel plot showing no publication bias in diastolic blood pressure.

This systematic review and meta-analysis had a number of notable strengths. Firstly, as per our knowledge, it is the first review study that assessed the effect of spinal manipulation and mobilization on cardiovascular responses. Second, all included studies were of high quality and had low risk of bias. Third, we only included RCTs which are considered gold standard in experimental studies. Further, no publication bias was observed from funnel plots (Figs. 4, 6 and 8). Despite these strengths, this study had certain limitations. Although we have communicated the corresponding authors of many studies to retrieve the missing data, but we have to exclude seven studies because of insufficient data. Moreover, we did only two database searches. More database searches would have increased the number of studies. Inclusion of more good quality studies from other databases might have increased our sample size and possibly reduced heterogeneity of the data. Heterogeneity is high for diastolic blood pressure and moderate for systolic blood pressure and heart rate in our meta-analysis. Hence, the results would have been more conclusive without these limitations.

**Fig. 7.**
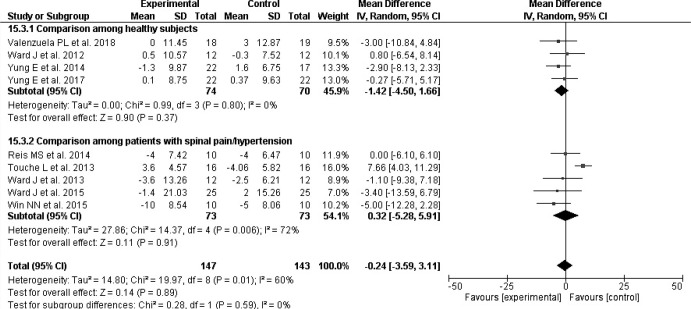
Comparison of heart rate using forest plot and subgroup analysis.

**Fig. 8.**
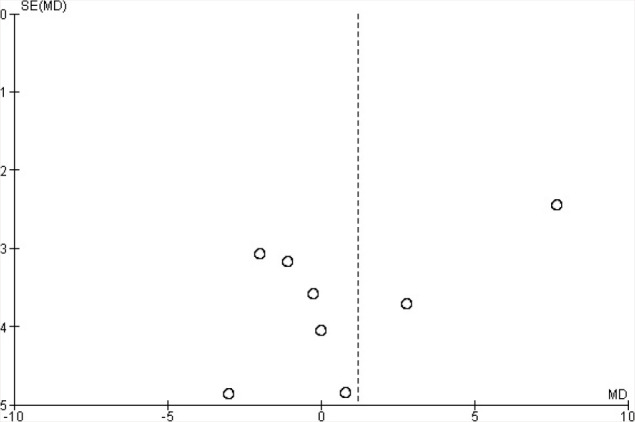
Funnel plot showing no publication bias in heart rate.

This systematic review and meta-analysis has a significant clinical implication. Spinal malalignments or spinal pain can be one of the most common causes of vertigo and secondary hypertension.^29^ Hypertension is becoming a serious problem nowadays. The lifetime risk of developing hypertension is approximately 90%.^30^ This may lead to serious complications such as coronary thrombosis, haemorrhage, stroke, heart attack, renal failure and so on. Patients with hypertension are prescribed antihypertensive drugs. There are a number of adverse effects of these antihypertensive drugs such as dizziness, hypotension, headache, flushing, nausea, peripheral edema, coughing, wheezing, pulmonary edema, acute lead to renal failure, dry cough, blurred vision, back pain, insomnia and many more. It may also produce muscle cramps and muscle weakness.^31^

Therefore, spinal manipulation and mobilization can be used as an adjuvant therapy in the treatment of patients suffering from hypertension with spinal malalignments. Manual therapy can reduce the drug dosage and dependency, thus preventing or decreasing drug-related adverse effects.

We have grouped together two different spinal manual therapy approaches that are spinal manipulations and mobilizations in our analysis. These techniques have different kinematics, biomechanics and mechanism of action. Therefore, the implications of our findings may not be ubiquitous. Results may vary depending on the type of technique and method of application. However, findings of the meta-analysis provide a preliminary evidence to support that spinal manual therapy including spinal manipulations and mobilizations may have a significant influence on cardiovascular parameters.

## Conclusion

Spinal manipulations and mobilizations showed significant reduction in SBP, but the heterogeneity was moderate. On sensitivity analysis, there was a significant decrease in both systolic as well as diastolic blood pressure and heterogeneity was also very low. Results were insignificant for heart rate. Therefore, within the limitations of the present systematic review and meta-analysis, it can be concluded that spinal manipulations and mobilizations may result in decrease of systolic as well as diastolic blood pressure. However, given the distinct kinematics, biomechanics and site of application of these techniques, more precise reviews can be performed to evaluate the efficacy of different types of manual therapy techniques applied at different regions of spine for progressing from generalized to specific conclusions.

## Conflict of Interest

The authors report no conflict of interest.

## Funding/Support

There was no funding source for this paper.

## Author Contributions

Manoj Malik and Charu Gera contributed to study conceptualization and design, data extraction and drafting of submitted paper. Jaspreet Kaur and Minaxi Saini contributed to analysis and interpretation of data as well as critical revisions in draft for important intellectual content.
